# Gender and Personality Differences in Response to Social Stressors in Great Tits (*Parus major*)

**DOI:** 10.1371/journal.pone.0127984

**Published:** 2015-05-26

**Authors:** Esther van der Meer, Kees van Oers

**Affiliations:** 1 Department of Animal Ecology, Netherlands Institute of Ecology (NIOO-KNAW), Wageningen, The Netherlands; 2 Cheetah Conservation Project Zimbabwe, Victoria Falls, Zimbabwe; Utrecht University, NETHERLANDS

## Abstract

In response to stressors, animals can increase the activity of the hypothalamic-pituitary-adrenocortical axis, resulting in elevated glucocorticoid concentrations. An increase in glucocorticoids results in an increase in heterophils and a decrease in lymphocytes, which ratio (H/L-ratio) is an indicator of stress in birds. The physiological response to a stressor can depend on individual characteristics, like dominance rank, sex and personality. Although the isolated effects of these characteristics on the response to a stressor have been well studied, little is known about the response in relation to a combination of these characteristics. In this study we investigate the relationship between social stress, dominance rank, sex and exploratory behaviour as a validated operational measure of personality in great tits *(Parus major)*. Great tits show consistent individual differences in behaviour and physiology in response to stressors, and exploratory behaviour can be classified as fast or slow exploring. We group-housed four birds, two fast and two slow explorers, of the same sex that were previously singly housed, in an aviary and compared the H/L-ratio, lymphocyte and heterophil count before and after group housing. After experiencing the social context all birds increased their H/L-ratio and heterophil count. Females showed a stronger increase in H/L-ratio and heterophil count than males, which seemed to be related to a higher number of agonistic interactions compared to males. Dominance rank and exploration type did not affect the H/L-ratio or heterophil count. Contrary to our expectations, all birds increased their lymphocyte count. However, this increase was slower for fast than for slow explorers. Our study suggests that personality and sex related differences, but not dominance rank, are associated with changes in an individual's physiological response due to a social context.

## Introduction

Throughout their lives, animals are subjected to various social and non-social stressors. Vertebrates have been reported to respond to such stressors with an increase in the activity of the hypothalamic-pituitary-adrenocortical (HPA) axis, which causes an increase in the concentration of circulating adrenal glucocorticoids (stress hormones) [1]. Whether or not a stressor provokes an increase in glucocorticoids depends on several factors, main factors being the predictability and controllability of the stressor [2]. When the HPA axis is activated, elevated levels of glucocorticoids ensure adequate energy supply to cope with the stressor [1,2]. However, if glucocorticoid levels remain elevated for an extended period of time it can result in harmful consequences, e.g. immunosuppression and weight loss [1,2]. Haematological measurements are widely used to assess an individual's stress level [3–5]. In birds the majority of white blood cells consist of heterophils and lymphocytes [3]. In response to elevated glucocorticoid levels, heterophil numbers increase [6], whereas lymphocyte numbers decrease [7,8], resulting in an increase of the heterophil to lymphocyte (H/L) ratio [9]. Consequently, the H/L-ratio has been widely used as an indicator of stress in birds [3].

For many species, social interactions and rank in the dominance hierarchy have important fitness consequences by affecting territory acquisition, mating success, reproduction and survival [10]. In order to establish and maintain dominance ranks animals engage in agonistic interactions [2]. These agonistic social interactions can be both unpredictable and uncontrollable and are known to be potent stressors [2,11]. The relationship between glucocorticoid concentrations, agonistic interactions and dominance rank is unclear. Some studies report that animals that lose fights (subordinates) show higher glucocorticoid levels than animals that win fights (dominants) [12,13], others demonstrate the contrary [14,15], whereas some find no difference [16,17].

The response to social stressors can be affected by dominance rank, but might also be affected by individual characteristics like sex [18–22] and personality [23–25]. Females may respond to social and non-social stressors with a higher glucocorticoid secretion compared to males [18–21]. It has been suggested that this difference is related to differences in sex steroids [19,20]. In addition, differences in personality are often characterized by consistent differences in behavioural, physiological and endocrine response to social and non-social stressors [23–27]. On a behavioural level, variation in animal personality has been shown to express itself in a variety of traits, e.g. shyness-boldness, exploratory behaviour, activity and aggressiveness [27]. Personality traits not only affect the outcome of social interactions and the establishment of dominance hierarchies [28–31], they also associate with an individual's glucocorticoid response to social stress [32–35]. Although several studies have studied the isolated effects of dominance rank, sex or personality on increasing glucocorticoid levels or H/L-ratios, the response in relation to a combination of these factors remains largely unknown.

In this study, we investigated the effect of social stressors, in relation to dominance rank, personality and sex, on haematological parameters (heterophil count, lymphocyte count, H/L-ratio) in captive groups of great tits. When defining personality it is important to study different behavioural traits [27], Réale et al. [27] propose to study personality within a framework that includes at least several of the five major behavioural trait categories they identified; exploration behaviour, shyness and boldness, activity, sociability and aggression. In great tits, individual differences in exploration behaviour have been shown to be related to differences in other behavioural traits like shyness and boldness [36], activity [37], sociability [26] and aggression [29]. Great tit personality has been found to range from bold, aggressive and fearless (hereafter referred to as fast explorers) to shy, less aggressive and fearful (hereafter referred to as slow explorers) [26,29,36,37]. Fast explorers are more aggressive but seem to have more difficulty to recover from defeat than slow explorers; after being repeatedly defeated by other individuals, it takes fast explorers a longer time to initiate a social interaction again [30]. We therefore expected the effect of dominance rank on changes in the H/L-ratio to be more pronounced for fast than for slow explorers, with subordinate fast explorers showing a higher response than dominant fast explorers. Females have been found to react more strongly to social stressors than males [21], we therefore expected females to show a stronger increase in H/L-ratio when experiencing a social context than males.

## Methods

### History and behavioural tests

Within this experiment we used 60 first year hand reared birds that originated from natural populations of great tits. At an age of ten days they were collected from the field and housed in sibling groups of 4–5 individuals in natural nests in cardboard boxes. The young were fed a mixed diet [37]. Survival during hand rearing was 95%. As soon as the birds started to leave the nest (17–20 days after hatching), sibling groups were housed in small wire cages of 0.5 x 0.4 x 0.4 m with two perches. From day 20 after hatching onwards, they were presented with a beef heart mixture supplemented with insects and hand rearing was gradually withdrawn. When the young could feed by themselves (25–30 days after hatching), they were housed individually in standard cages of 0.9 x 0.4 x 0.5 m with a solid bottom, top, sides and rear walls, a wire mesh front and three perches. The birds were kept under natural light conditions and had auditory and visual contact with other individuals. They were provided with *ad libitum* drinking water, sunflower seeds and commercial seed mixture, supplemented with a protein-rich mixture [38].

Two days after individual housing the birds were subjected to standard exploration tests developed by Verbeek et al. [37]: a novel environment test and two tests measuring the response to different novel objects (see also [24,26,29,34,35,37,38]). The novel environment test was conducted in an observation room of 4.2 x 2.5 x 2.3 m with five artificial trees. The bird's home cages were connected to the observation room by a sliding door. To let the birds enter the observation room without being handled, their home cage was darkened with a towel making the birds fly into the observation room when the light was switched on. We measured the time within which a bird visited four out of five artificial trees. To calculate exploratory tendency this time was transformed into a linear scale ranging from 0 (bird did not visit four trees within 10 min) to 10 (bird visited four trees within 1 min).

Respectively, seven and nine days later this test was followed by novel object tests in which the birds were presented with a penlight battery (day 7) and a pink panther toy (8 cm) (day 9). The novel object was placed in the cage on one of the outer perches. The time it took the bird to approach this perch and the shortest distance to the novel object within 120 s were used to assess a score, ranging from 0 (the bird did not land on the perch with the object) to 5 (the bird pecked the object). The sum of the three test scores (0–20) was used to classify the birds, with 0 being the most extreme slow explorer and 20 being the most extreme fast explorer [38]. Birds that were quick to visit the artificial trees were also quick to approach the novel objects (see also [37]). For this experiment, we selected birds that were identified as either fast (scores > 6) or slow explorers (scores < 4) based on the 25% and 75% quartiles of tests on 1000 animals.

Three days before the start of the experiment, birds were captured by hand from their individual cage for blood sampling and to determine moult score, body mass and tarsus length. Moult score was based on the last moulted primary feather (score 1–10). Body mass was measure in grams, tarsus length in millimetres. Blood samples of 10 μl were taken from the brachial vein to determine sex, using molecular markers as described by Griffiths [39], and to determine the initial H/L-ratio, lymphocyte and heterophil count in the non social home cage context before the experiment. Each bird was fitted with three coloured leg rings to enable individual identification. Within each experimental flock the assignment of ring colour was randomised in order to avoid a possible behavioural bias related to ring colour [40,41].

### Behavioural observations

Three days after blood collection, birds were captured by hand from their home cages and simultaneously released into semi-open aviaries of 4 x 2 x 2 m with a wire-mesh front and top, six branches and six nest boxes. In total we formed 15 uni-sex flocks of four birds, eight male flocks (n = 32) and seven female flocks (n = 28). Each flock consisted of four birds; two birds characterised as fast explorers (♂ n = 16, ♀ n = 14), and two birds characterised as slow explorers (♂ n = 16, ♀ n = 14). The experiment lasted seven days. During these days the birds were kept under natural light conditions and were provided with *ad libitum* drinking water, sunflower seeds and commercial seed mixture, supplemented with a protein-rich mixture. After releasing the four birds in the aviary on the first day, behavioural observations were made for seven subsequent hours. On the second day, flocks were observed for three hours (one hour observation periods in the morning, afternoon and late afternoon). From the third until the seventh day, flocks were observed for forty-five minutes (fifteen minute observation periods in the morning, afternoon and late afternoon). In order to increase the number of interactions between the birds, we provided the flocks with greater wax moth larvae *(Galleria mellonella)* during observations on the 2nd until the 7th day. Antagonistic interactions between individual birds (i.e. one individual displacing another individual, avoidance, threatening, fighting) were recorded using a winner-loser matrix [42,43]. Verbeek et al. [30] showed it takes on average seven days before a stable hierarchy develops among a group of aviary great tits. Based on the outcome of the observed antagonistic interactions, we determined an individual's dominance rank in the hierarchy on the seventh day. At the end of the experiment, each bird was captured from its aviary with a net and taken outside the aviary in order to take blood samples from the brachial vein, and to determine body mass and moult score.

### Blood sampling and cell count

When captured from its aviary, the bird was hand held on its back with the thumb placed loosely over the body, and fixed by keeping its head and the tip of the left wing between the index and middle finger. A drop of blood was collected from the brachial vein, and immediately smeared onto a slide. Each bird was captured and blood sampled within < 3 minutes. All blood samples and biometric measurements were taken between 14h and 16h.

Smears were air dried, fixed with 96% methanol and stained with Hemacolor (Merck KgaA). The smears were screened using a light microscope with 10x ocular and 100x oil immersion lenses. A 1.0 x 1.0 cm grid was used to count the number of heterophils and lymphocytes per 10 000 erythrocytes. Based on this count we calculated the H/L-ratio by dividing the number of heterophils by the number of lymphocytes.

### Statistical analyses

A Chi-square test was used to test for differences in the number of fast and slow exploring birds that held dominant, intermediate or subordinate ranks in the dominance hierarchy seven days after release in the aviary. We used a two-tailed t-test to test for differences in the number of interactions between males and females and fast and slow exploring individuals. A two tailed paired t-test was also used to test for differences in haematological values before and after the experiment.

We used general linear mixed-effect models to test for differences in lymphocyte count, heterophil count, H/L-ratio, body mass, tarsus length and moult score before the experiment. Sex, exploration type of the bird, and the interaction between sex and exploration type were added to the model as explanatory variables. We also used linear mixed models to test for difference in lymphocyte count, heterophil count, H/L-ratio, and body mass after the experiment. After the experiment all birds had completed moult, moult score was therefore left out of these analyses. Sex and exploration type of the bird, its dominance rank at the end of the experiment, all 2-and 3-way interactions between these factors, and the respective blood count (lymphocyte count, heterophil count or H/L-ratio) before the experiment were added to the model as explanatory variables. To identify the variables affecting haematological parameters and body mass, we used a backward stepwise selection procedure based on the restricted maximum likelihood ratio with successive removal of non-significant variables. We removed terms for which p > 0.05, starting with interactions, until the model only contained significant terms (minimal adequate model). In cases where the t-tests showed a difference in the number of interactions, we ran the minimal adequate linear mixed effect model controlling for the number of interactions to see if a sex or exploration type difference remained. To account for non-independence of the data, i.e. repeated measures from individuals in the same aviary, we included individual nested in aviary as a random variable in all mixed models. For post-hoc analysis, we used a Tukey test to assess differences in lymphocyte count before the experiment between male and female fast and slow explorers. All statistical analyses were performed using SPSS software version 20.0 (SPSS Inc., Chicago, IL, U.S.A.).

### Ethical note

For this study, a small blood sample was taken twice from the brachial vein. To minimize the potential effect on the health of the birds, we used the right wing for the sample before the experiment and the left wing for the sample after the experiment. During social hierarchy measurements birds showed ritualized agonistic behaviour. Most attacks were 'supplanting' attacks without physical contact. In rare cases where physical contact occurred this contact did not result in escalated fights or injuries. No injuries were observed caused by blood sampling or agonistic interactions with conspecifics, nor did any of the birds die during the experiment. All birds gained weight during the seven days in the aviary (mean ± SE = 2.60 ± 0.19 g).

Het Gelders Landschap en Kastelen, provided permission to collect the birds from their land. The birds were housed and subjected to the experiment at the Netherlands Institute of Ecology (NIOO-KNAW), department of Animal Ecology. After the experiment, birds were either used to form breeding pairs that were housed in aviaries at the NIOO-KNAW or, when unnecessary for other research purposes, released into the wild in a controlled reintroduction programme. The collection of birds from the field, the experiment as described in this manuscript and the housing and breeding of the birds after the experiment were approved by the animal ethical committee of the Royal Dutch Academy of Science (DEC-KNAW) under license number CTO99-01 to KVO.

## Results

### Body mass, moult score and haematology before the experiment

**Fig 1:**
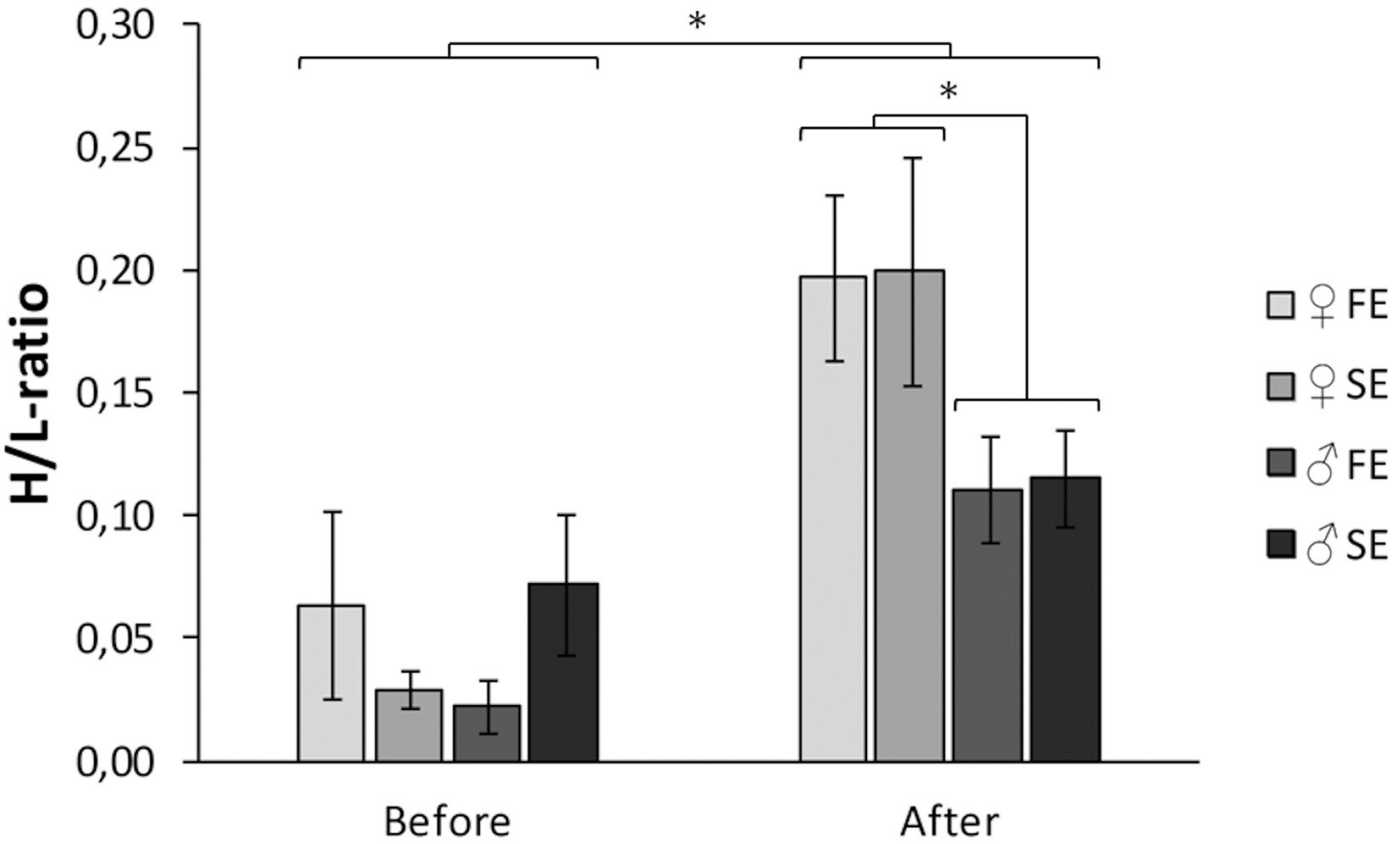
H/L-ratio of male (♂) and female (♀) fast (FE) and slow exploring (SE) great tits before, and seven days after experiencing the social context. Vertical bars represent one standard error of the mean. Asterisk denotes significant differences between groups.

**Fig 2:**
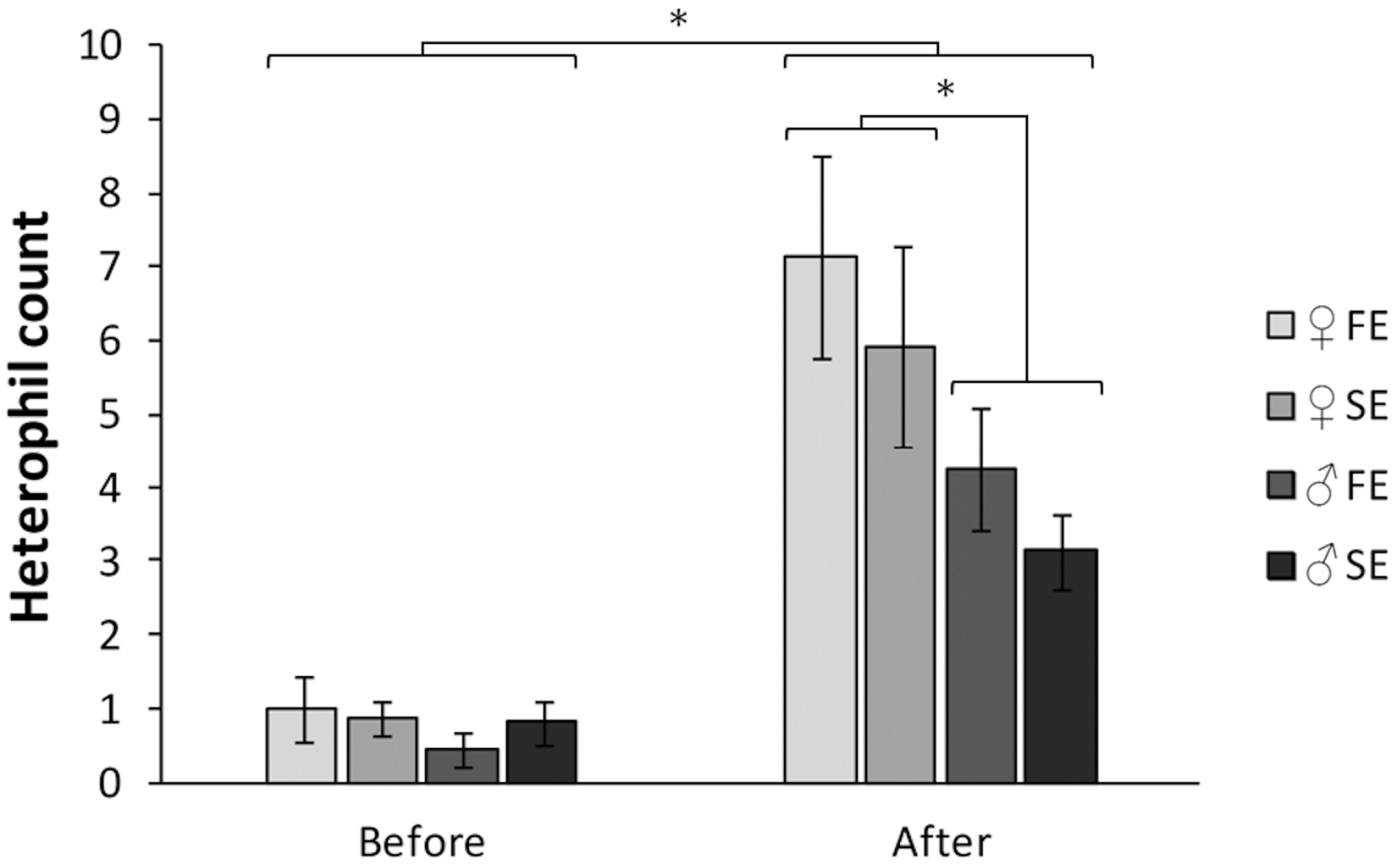
Heterophil count of male (♂) and female (♀) fast (FE) and slow exploring (SE) great tits before, and seven days after experiencing the social context. Vertical bars represent one standard error of the mean. Asterisk denotes significant differences between groups.

**Fig 3:**
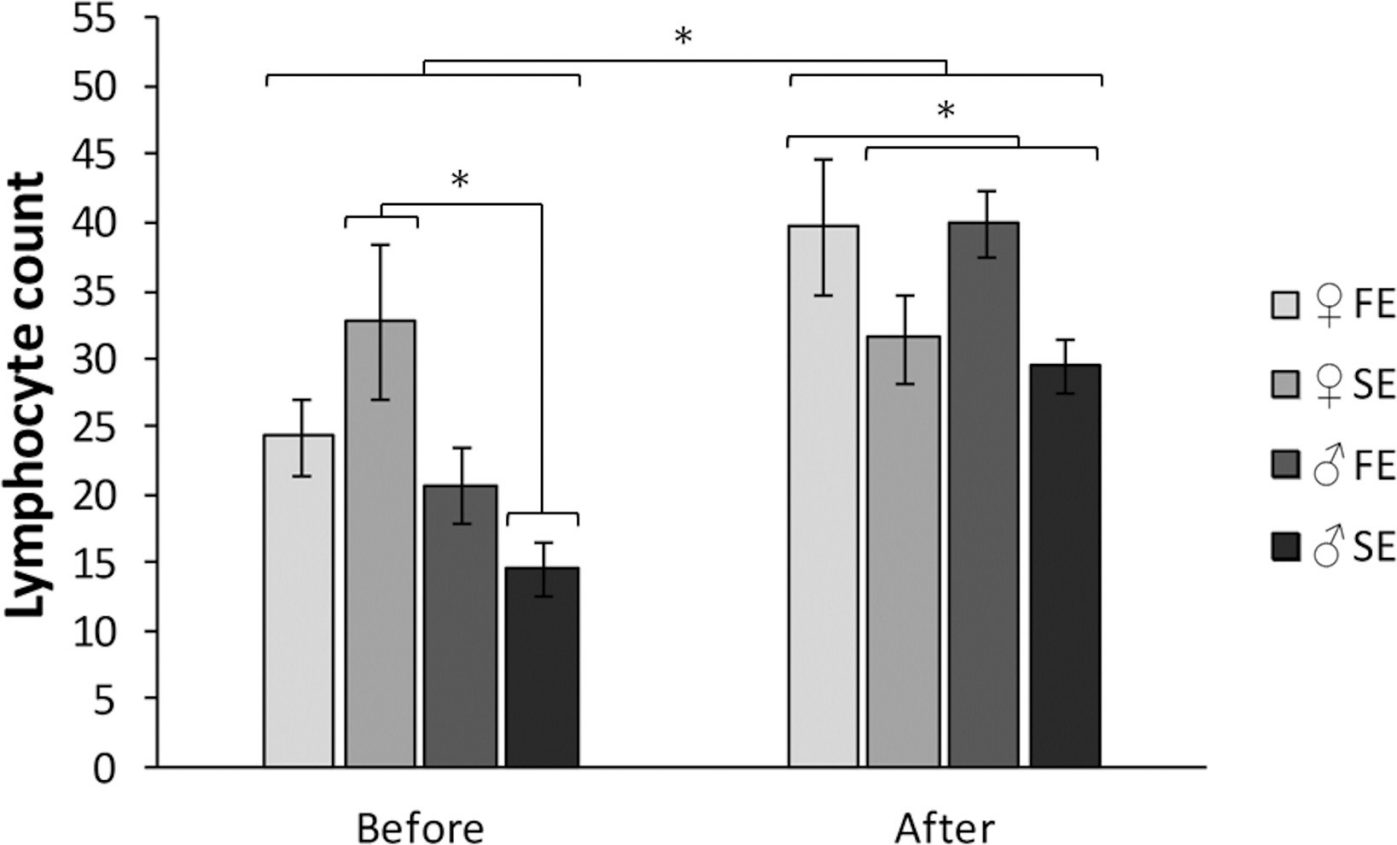
Lymphocyte count of male (♂) and female (♀) fast (FE) and slow exploring (SE) great tits before, and seven days after experiencing the social context. Vertical bars represent one standard error of the mean. Asterisk denotes significant differences between groups.

Controlling for sex differences, body mass was not affected by exploration type (F_1, 57_ = 2.09, *P* = 0.15), neither was tarsus length (F_1, 57_ = 0.52, *P* = 0.47) or moult score (F_1, 57_ = 1.19, *P* = 0.28). There were no differences in H/L-ratio or heterophil count between males and females or fast and slow explorers (all *P* > 0.33; Figs 1 and 2). We did find a sex dependent effect of exploration type on the lymphocyte count (exploration type x sex interaction; F_1, 56_ = 4.46, *P* = 0.04). This was caused by a sex difference in the slow exploring type: slow exploring females showed a significantly higher lymphocyte count than slow exploring males (Tukey test statistic, *P* < 0.01; Fig 3).

### Dominance rank and agonistic interactions after the experiment

**Fig 4:**
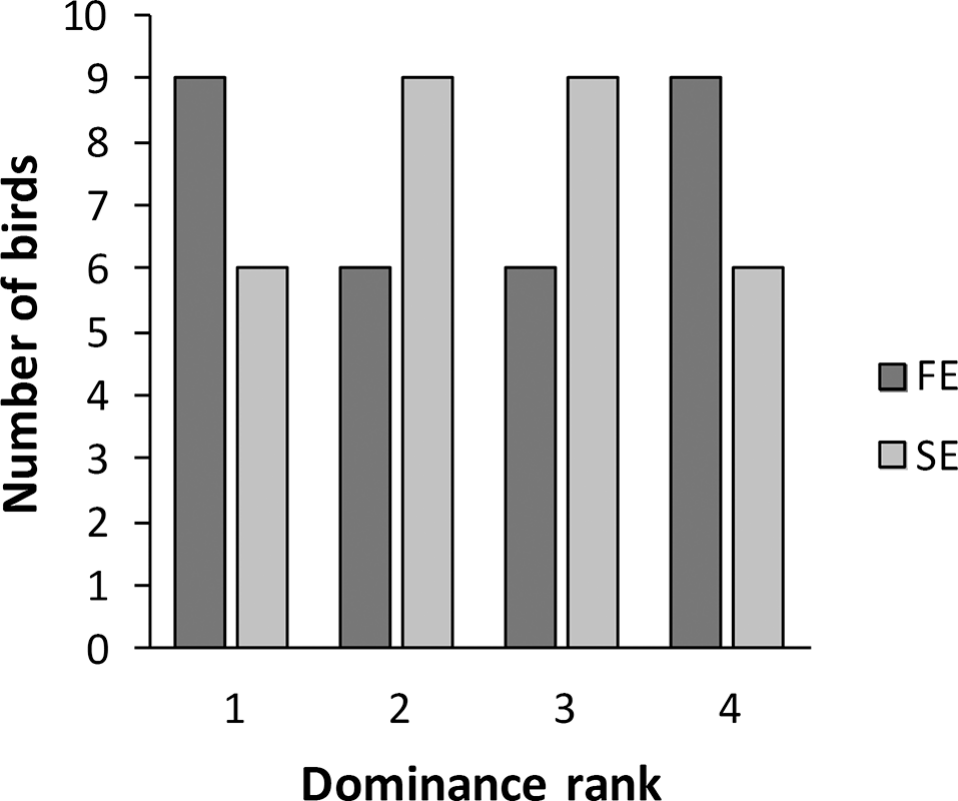
Number of fast exploring (FE) and slow exploring (SE) birds at the different ranks in the dominance hierarchy seven days after release in the aviary.

We found no difference in dominance rank between fast and slow explorers seven days after release in the aviaries (χ^2^ = 2.40, df = 3, *P* = 0.49; Fig 4). The total number of interactions during the 7 days in the aviary was higher for females than for males (t_58_ = - 4.04, *P* < 0.01, ♀ mean ± SE = 231.36 ± 11.97, ♂ mean ± SE = 163.88 ± 11.60). There was no difference in the total number of interactions between fast and slow exploring birds (t_58_ = 0.31, *P* = 0.76, FE mean ± SE = 198.30 ± 13.27, SE mean ± SE = 192.43 ± 13.39).

### Body mass, moult score and haematology after the experiment

**Fig 5:**
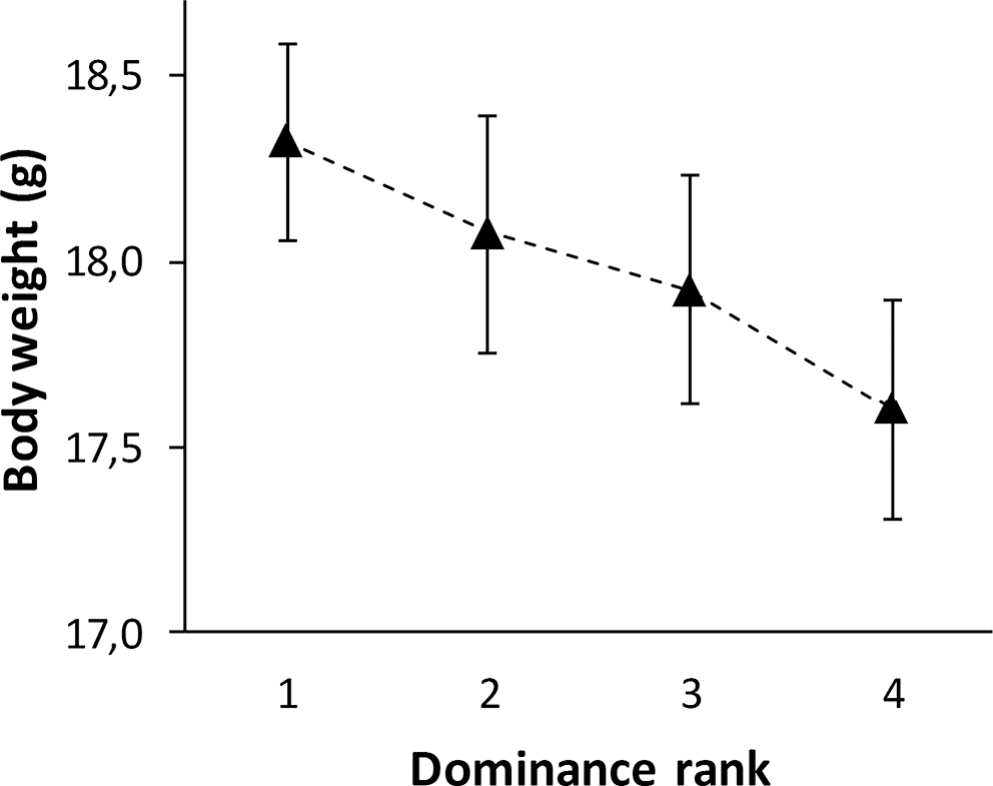
Body mass in relation to dominance rank seven days after release in the aviary. Vertical bars represent one standard error of the mean.

Body mass was affected by rank in the dominance hierarchy after controlling for sex differences in body mass (F_3,55_ = 3.29, *P* = 0.03; Fig 5), with dominant individuals being heavier than subordinate individuals (β ± SE = 0.72 ± 0.24, *P* < 0.01; Fig 5).

We found a positive relationship between the H/L-ratio before the experiment and the H/L-ratio after the experiment (β ± SE = 0.38 ± 0.16, *P* = 0.02). When controlling for H/L-ratio differences before the experiment, the H/L-ratio after the experiment was not affected by exploration type (F_1,55_ = 0.00, *P* = 1.00) or dominance rank (F_3,54_ = 0.32, *P* = 0.81). We did find a difference between males and females (*P* < 0.01): after the experiment males showed a lower H/L-ratio than females (β ± SE = -0.09 ± 0.03; Fig 1). This sex difference disappeared when controlling for the number of interactions (*P* = 0.18), suggesting a direct relationship between the number of interactions and the H/L-ratio. For both males and females H/L-ratio after seven days in a social context was higher than in the non-social context before the experiment (♂ t_31_ = -3.27, *P* < 0.01, ♀ t_27_ = -5.74, *P* < 0.01; Fig 1).

Heterophil count was positively related to the heterophil count before the experiment (β ± SE = 1.39 ± 0.41, *P* < 0.01). When controlling for the heterophil count before the experiment, heterophil count was neither affected by exploration type (F_1,56_ = 2.04, *P* = 0.16) nor dominance rank (F_3,53_ = 0.07, *P* = 0.98). There was a difference between the sexes: the heterophil count after the experiment was lower for males than for females (β ± SE = - 2.43 ± 0.97, *P* = 0.02; Fig 2). This difference between males and females disappeared when controlling for the number of interactions (*P* = 0.22). For both males and females heterophil count after the experiment was higher than before the experiment (♂ t_31_ = -5.94, *P* < 0.01, ♀ t_(27)_ = -6.68, *P* < 0.01; Fig 2).

Lymphocyte count after the experiment tended to be positively related to the lymphocyte count before the experiment (β ± SE = 0.21 ± 0.11, *P* = 0.05). When correcting for this effect we found an effect of exploration type: after the experiment fast exploring birds showed a higher lymphocyte count than slow exploring birds (β ± SE = 9.59 ± 3.08, *P* < 0.01; Fig 3). Dominance rank (F_3,54_ = 0.89, *P* = 0.45) and sex of the bird (F_(1,53)_ = 0.17, *P* = 0.68) did not affect lymphocyte count after the experiment. For both fast explorers and slow explorers lymphocyte count after the experiment was higher than before the experiment (fast explorers t_29_ = -5.86, *P* < 0.01, slow explorers t_29_ = -2.38, *P* = 0.02; Fig 3).

## Discussion

In this study we show that regardless of dominance rank, sex and personality, the H/L-ratio increases when great tits were presented with social stressors, indicating that group living in an aviary is more stressful compared to single housing in a home cage.

The relationship between glucocorticoid levels and rank in the dominance hierarchy varies [12–17]. Differences in personality have been found to affect an individual's dominance rank [30,44,45] and the ability to cope with defeat [30]. In this study, the observed increase of the H/L-ratio after experiencing social stressors did not seem to be related to dominance rank and/or personality. In the wild, great tits are territorial and establish, site dependent, stable linear dominance hierarchies [46–48]. Within an aviary set-up linear hierarchies develop but, although after the initial two days the number of dominance shifts decrease to < 10%, dominance shifts never reach zero [30]. Glucocorticoid levels have been reported to be higher when hierarchies are unstable [49,50]. In addition, acute exercise has been shown to increase glucocorticoid secretion [51,52]. It is therefore likely that increased levels of glucocorticoids in response to social stressors are not only related to the effects of defeat and replacement but also to the acute physical activity required when engaging in agonistic interactions. The complex relationship between glucocorticoid levels, the level of instability within dominance ranks and the physical activity related to agonistic interactions, could potentially have masked an effect of dominance rank in relation to personality. In accordance with other studies [53], we did find an effect of dominance rank on body mass, with dominant birds being heavier than subordinate birds, which could be the result of better access to food resources of dominant individuals [54] and/or higher glucocorticoid levels of subordinate individuals [55].

Contrary to our expectation, all birds showed an increase in lymphocyte count after experiencing the social context. Lymphocytes are involved in regulating the immune response and eliminating antigens, and increase in response to immunological challenges (e.g. [56–58]). It is possible that, compared to the individual housing before the experiment, during group housing in semi-open aviaries, the birds got exposed to a new set, or more, immunological challenges that resulted in an increase in the number of lymphocytes. For fast exploring birds this increase was stronger than for slow exploring birds. This difference in increase might be related to differences in glucocorticoid levels between fast and slow explorers. In response to a stressor, fast exploring individuals show higher neurosympathetic activity and low glucocorticoid levels, while slow explorers show high cardiac parasympathetic activity and high glucocorticoids levels [23,32–35]. Elevated levels of glucocorticoids result in a decrease in the number of blood lymphocytes [8]. When being exposed to social stressors it is possible that slow exploring birds experience higher levels of glucocorticoids, which reduce the number of lymphocytes, resulting in a smaller increase in lymphocyte count compared to fast explorers. In addition, in great tits, slow explorers have been found to display higher levels of testosterone than fast explorers [59]. Elevated levels of testosterone have been linked to a reduced lymphocyte count [60,61]. Differences in testosterone levels between males and females [62] and fast and slow exploring birds [59] might therefore not only explain the higher lymphocyte count of slow exploring females compared to slow exploring males before the experiment but could also play a role in the differences in lymphocyte count between fast and slow explorers after experiencing the social context.

The H/L-ratio and heterophil count after experiencing the social context were affected by the individual's sex. Although both sexes showed an increase in H/L-ratio and heterophil count, females showed a stronger increase than males, suggesting a higher level of glucocorticoids. These differences seem to be related to a difference in the number of interactions between males and females. Although in the wild, male-male interactions are more common than female-female interactions [44], in our study females engaged in a higher number of interactions than males. Unlike for males, in a wild situation, the outcome of female-female interactions depends on the presence of the mate [46]. Although this suggests the mate plays an important role in female-female aggression, it is unknown how the presence of the mate affects the number of interactions between female great tits. In other species it was found that the absence of the mate increased the number of female-female interactions [63]. In our study, the absence of mates could possibly explain the relatively high number of female-female interactions which seems to have resulted in a stronger increase in the H/L-ratio and heterophil count of females compared to males. Several studies have demonstrated a sex difference in response to stressors [18–22], with females showing a higher glucocorticoid secretion than males [21], which in turn can result in a higher heterophil count [6]. This difference in glucocorticoid secretion seems to be related to differences in sex steroids [19,20]. However, in our study the difference in H/L-ratio and heterophil count disappeared when controlling for the number of interactions. This suggests that, with acute exercise resulting in an increase in glucocorticoid secretion [51,52], the found sex differences are likely to predominantly be the result of differences in the number of interactions.

Handling has been shown to evoke an acute stress response in great tits [24,64]. Although in response to handling stress, birds increase their glucocorticoid concentrations within 1–3 minutes after capture [35,65–67] this is unlikely to have affected our haematological parameters. Studies on rats show that, in response to restraint, haematological parameters change after 6 minutes [66]. Avian studies show that up to a handling time of 30 minutes, handling stress does not affect haematological parameters [4,68]. Our short handlings times (< 3 min) are therefore unlikely to have affected the haematological values as presented in this study. In addition, the birds are likely to have experienced the same handling stress during blood sampling before and after the experiment, enabling a reliable comparison of changes in haematological parameters after experiencing the social context.

In accordance with other (comparable) studies [69–71], the individual birds in our study served as their own control. It is possible that during the experiment the birds were exposed to stressors other than social stressors. The most likely additional stressor being the exposure to a novel environment. Exposure to a novel environment has been shown to result in increased levels of glucocorticoids [72]. However, several studies show that when animals are continuously or repeatedly exposed to a novel environment, habituation occurs [73]. Habituation due to continued exposure typically occurs within minutes after entering the novel environment [74–77]. The seven days in the aviary prior to blood sampling should therefore be long enough to exclude any additional effects from exposure to this novel environment. Alternatively, seasonal variation in haematological parameters might have played a role in our study. Although some studies found no effect of season [78], others show that heterophil count, lymphocyte count and H/L-ratio show seasonal variation (e.g. [79–81]). Great tits have been found to show seasonal variation in haematological parameters related to moult; with a peak in heterophil count and H/L-ratio during the early moulting period followed by a fast decrease during the second half of the moulting season [80]. The birds in our experiment all completed moult during the seven days in the aviary. If seasonal variation related to moult had affected our haematological parameters we should have found a decrease in H/L-ratio rather than an increase. It is therefore more likely that the changes in haematological parameters as found in this study were related to social stressors.

In summary, this study shows that personality and sex related differences, but not dominance rank are associated with differences in an individual's haematological response to social stressors. As expected, females responded with a stronger increase in heterophils and elevated H/L-ratio's compared to males, which seems to be related to a higher number of antagonistic interactions between females. Although lymphocyte count increased in the social context in both exploration types, slow exploring individuals seemed to have a reduced increase in the number of lymphocytes compared to fast explorers. This difference might be explained by the predominantly sympathetic response to stressors of fast explorers and parasympathetic response of slow explorers [23,32–35]. In conclusion, our study shows that both gender and personality can affect an individual's response to social stressors, and underlines the importance of taking this into account in future studies on stress in social animals.
